# Laser-assisted wet coating of calcium phosphate for surface-functionalization of PEEK

**DOI:** 10.1371/journal.pone.0206524

**Published:** 2018-10-31

**Authors:** Ayako Oyane, Maki Nakamura, Ikuko Sakamaki, Yoshiki Shimizu, Saori Miyata, Hirofumi Miyaji

**Affiliations:** 1 Nanomaterials Research Institute, National Institute of Advanced Industrial Science and Technology (AIST), Tsukuba, Ibaraki, Japan; 2 Department of Periodontology and Endodontology, Faculty of Dental Medicine, Hokkaido University, Sapporo, Hokkaido, Japan; University of South Carolina, UNITED STATES

## Abstract

Calcium phosphate (CaP) coating is an effective method for surface-functionalization of bioinert materials and for production of osteoconductive implants. Recently, we developed a laser-assisted biomimetic process (LAB process) for facile and area-specific CaP coating. In this study, the LAB process was applied to chemically stable and mechanically durable poly(etheretherketone) (PEEK), which has become widely used as an orthopedic and dental implant material. The LAB process was carried out by irradiating pulsed Nd:YAG laser light (355 nm) onto a PEEK substrate that was immersed in supersaturated CaP solution. The CaP coating applicability depended on laser fluence, *i*.*e*., CaP successfully formed on PEEK surface after the LAB process at 2 W/cm^2^. Further increase in laser fluence did not result in the successful formation. At the optimal fluence of 2 W/cm^2^, the laser-irradiated PEEK surface was modified and heated to induce heterogeneous CaP precipitation within 10 min in CaP solution, followed by further CaP growth over the irradiation time (tested up to 30 min). The LAB process improved the cytocompatibility of PEEK surface with osteoblastic MC3T3-E1 cells. Furthermore, the LAB-processed CaP-coated PEEK substrate formed a dense hydroxyapatite layer on its surface in the simulated body fluid, suggesting the osteoconductivity of this material. The present LAB process can be a useful new tool to produce osteoconductive PEEK-based implants.

## Introduction

Calcium phosphate (CaP) is the main inorganic constituent of human bone tissue. CaP-based ceramics, including sintered hydroxyapatite and β-tricalcium phosphate, have been widely used as implants for bone tissue replacement, because they can bond to and integrate with living bone tissues (the property known as osteoconductivity) [1, 2]. However, these CaP-based ceramics cannot be used under high load-bearing conditions because of their insufficient fracture toughness.

A super engineering plastic of poly(etheretherketone) (PEEK) has become a popular polymeric material used for orthopedic and dental implants because it exhibits high chemical stability and mechanical properties suitable for bone tissue replacement [3]. Different from metallic implants, PEEK-based implants are radiolucent and have few concerns over the risk of stress shielding and allergenic reactions. Despite such useful characteristics, PEEK is intrinsically bioinert. Therefore, there is a growing need for osteoconductive PEEK-based implants [3].

To date, various approaches, such as CaP coating [4–9], incorporation of osteoconductive ceramic fillers [10–12], and surface modification [13–17], have been proposed to alter bioinert PEEK surface into the osteoconductive one [18–20]. Among these approaches, a biomimetic CaP coating process [5, 6, 9], which uses supersaturated CaP solution as a reaction medium, has drawn increasing attention because of the recent trend toward multifunctional CaP coatings combined with bioactive substances [21, 22]. In a typical biomimetic process, a substrate of artificial materials is first surface-modified with specific functional groups and/or CaP seeds (surface modification step). The surface-modified substrate is subsequently immersed in an acellular and metastable supersaturated CaP solution for further CaP growth (immersion step). By adding certain bioactive substances (proteins, DNAs, trace elements, etc.) to the CaP solution during the immersion step, these substances are immobilized within the resulting CaP coatings through coprecipitation with CaP [23].

Conventional biomimetic processes are weak in productivity, which require a fairly long processing period (more than a day) because of multi-step procedures and slow CaP growth rate in the immersion step. Recently, we combined a laser process with a biomimetic process to achieve facile single-step CaP coatings on several artificial materials [24–28] by refining earlier techniques proposed by Pecheva *et al*. [29] and Lee *et al*. [30]. In our laser-assisted biomimetic process (LAB process), a weak pulsed laser is used without focusing to irradiate a substrate that is immersed in a supersaturated CaP solution. Coating of CaP is accomplished in one-pot in this single-step procedure within tens of minutes. In addition, it is capable of realizing area-specific CaP coating because the coating area is confined to the laser-irradiated region of the substrate surface. These are noteworthy advantages of the LAB process over conventional biomimetic processes.

The aim of this study is to apply the LAB process for CaP coating to PEEK substrates and to demonstrate the improved biological properties of the resulting material. The LAB process has been applied to several artificial materials with laser absorption [24–28]. However, its applicability to chemically unreactive PEEK remains to be clarified. In this study, PEEK substrates were subjected to the LAB process under various conditions using a supersaturated CaP solution (so-called CP solution [31]). The mechanism of CaP formation on the LAB-processed PEEK substrate was examined by a time course study along with two control experiments. Cytocompatibility of the LAB-processed PEEK substrate was evaluated using osteoblastic MC3T3-E1 cells. For the preliminary osteoconductivity assessment, the LAB-processed PEEK substrate was immersed in a simulated body fluid (SBF) [32] with ion concentrations close to those of human blood plasma. The SBF test has been used to predict *in vivo* osteoconductivity of a material, *i*.*e*., a material which forms a hydroxyapatite layer on its surface in SBF is expected to bond to living bone tissues through the *in vivo* apatite formation at the bone-material interface [32].

## Materials and methods

### Preparation of PEEK substrates and CP solution

A 3-mm thick plate of PEEK (VICTREX PEEK Polymer 450G) was generously supplied by Victrex Japan Inc., Japan. The PEEK plate was cut into substrates with a dimension of 30 mm × 30 mm (for light absorption measurement), 4 mm × 4 mm (for cytocompatibility assessments), or 10 mm × 10 mm (for all other experiments). The substrates were polished on one side with #2000 SiC abrasive paper (*ϕ* = 15–18 μm) and washed ultrasonically with ethanol. The substrates were dried in air and stored in an electric desiccator before use, and are referred to as “untreated substrates”.

The CP solution was prepared by the method described elsewhere [23, 31]. Briefly, reagent grade chemicals (all from Nacalai Tesque, Inc., Japan) of NaCl (final concentration = 142 mM), K_2_HPO_4_·3H_2_O (1.50 mM), 1M HCl (40 mM), and CaCl_2_ (3.75 mM) were dissolved into ultrapure water sequentially, and buffered to pH = 7.40 at 25.0°C with tris(hydroxymethyl)aminomethane (50 mM) and a necessary amount of 1M HCl. The as-prepared CP solution was sealed in a polystyrene bottle and stored in a refrigerator (4°C) before use (used within a month).

### LAB process for CaP coating

**Fig 1:**
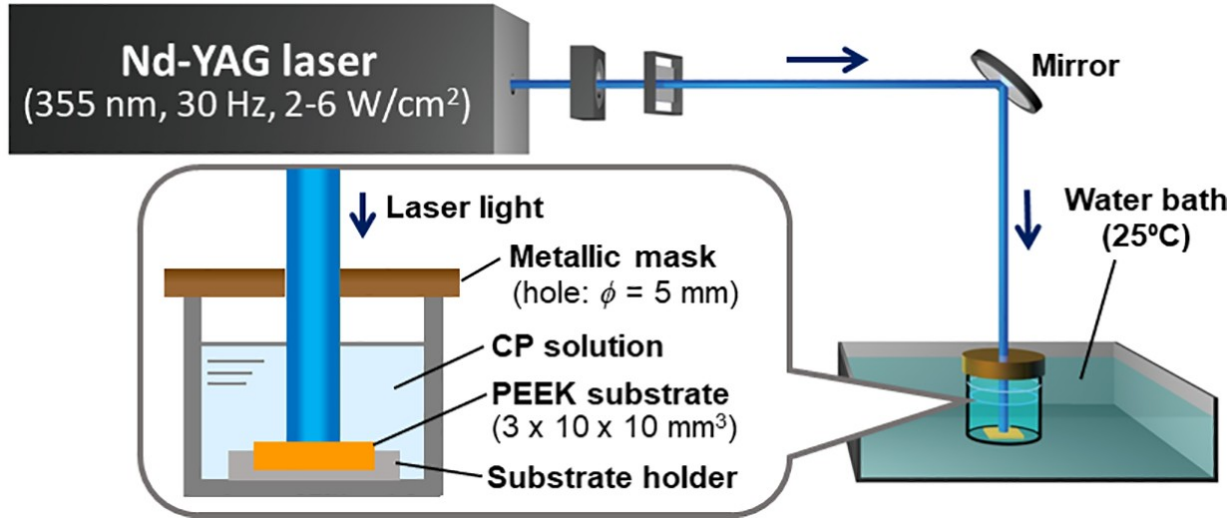
Schematic of the irradiation system of the LAB process for the standard-sized PEEK substrate (3 mm × 10 mm × 10 mm).

Fig 1 shows the irradiation system of the LAB process for the standard-sized PEEK substrate (10 mm × 10 mm surface). First, one PEEK substrate was fixed in a poly(tetrafluoroethylene) substrate holder that was then placed at the bottom of a glass bottle. The CP solution (10 mL) was added to the bottle, and the substrate was fully immersed in it. The glass bottle was placed in a water bath at 25°C. The PEEK substrate surface was irradiated with pulsed ultraviolet (UV) light (λ = 355 nm) using a Nd:YAG laser (Quanta-Ray LAB-150-30, Spectra-Physics, USA) operating at 30 Hz without focusing. The laser beam was passed through a 5-mm diameter hole of a metallic mask to irradiate a circular region (*ϕ* = 5 mm) onto the PEEK surface. For the smaller PEEK substrate (4 mm × 4 mm surface) for cytocompatibility assessments, the metallic mask was not used, thus the entire surface was irradiated. The output energy density (fluence) of the laser beam was adjusted at 2, 4, or 6 W/cm^2^ (67, 133, or 200 mJ/pulse/cm^2^, respectively). After irradiation for various periods up to 30 min, the substrate was removed from the CP solution and gently rinsed with ultrapure water. The CP solution remained clear after the LAB process without inducing homogeneous CaP precipitation.

### Control experiments

To elucidate the surface reactions during the LAB process, two control experiments were carried out. First, the LAB process was performed for PEEK substrates without a water bath to estimate the photothermal effect. After laser irradiation for various periods up to 30 min, the solution temperature was measured using a commercial thermometer. For comparison, the solution temperature without PEEK substrates (only the substrate holder was placed in the bottle) was also measured. Second, laser irradiation was applied to PEEK substrates using ultrapure water as the reaction medium in place of the CP solution to examine surface structural changes of the bare substrate without CaP.

### Physicochemical analysis

Light absorption of the untreated PEEK substrate was examined using an ultraviolet-visible-near-infrared (UV-VIS-NIR) spectrophotometer (SolidSpec-3700 DUV, SHIMADZU Corporation, Japan). The large substrate of 3 mm × 30 mm × 30 mm was used to fully cover the analytical window.

The substrate surface was analyzed using a scanning electron microscope (SEM; Model XL30, FEI Company, USA and S-4800, Hitachi High-Technologies Corporation, Japan) equipped with an energy-dispersive X-ray (EDX) analyzer (Genesis 2000, AMETEK, Inc., USA and EMAX x-act, HORIBA, Ltd., Japan), a digital camera (COOLPIX P330, Nikon Corporation, Japan), and a contact angle meter (Drop Master DM300, Kyowa Interface Science, Co. Ltd., Japan). Before SEM-EDX analyses, the substrates were mounted on a metallic sample holder, vacuum dried at room temperature overnight and then coated with conductive carbon using a carbon coater (VC-100, Vacuum device, Co., Ltd., Japan). In the contact angle measurements, an image of the water drop was captured 1 s after a droplet (1 μL) of ultrapure water contacted with the substrate surface.

The CaP precipitate formed by the LAB process under the selected condition (2 W/cm^2^, 30 min) was analyzed using a transmission electron microscope (TEM; JEM-2010, JEOL Ltd., Japan) equipped with an EDX analyzer (Genesis RTEM-S, AMETEK, Inc., USA). Before TEM analysis, the precipitate was gently scraped using a stainless steel spatula from the LAB-processed PEEK substrate and mounted directly onto a carbon-coated copper grid (HRC-C10, Okenshoji Co., Ltd., Japan). The TEM images and transmission electron diffraction (TED) patterns of the precipitate were obtained at an accelerating voltage of 200 kV. In the TED analysis, the camera length required for the d-spacing calculation was calibrated using a thallium chloride film, which is a standard calibration sample. Two different portions of the precipitate were analyzed.

### *In vitro* test for cytocompatibility assessment

Mouse osteoblastic MC3T3-E1 cells (RIKEN BioResource Center, Japan) were used for quantitative and qualitative cytocompatibility assessments. The cells were cultured in a minimum essential medium (αMEM; α-GlutaMAX-I, Thermo Fisher Scientific, USA) that was supplemented with 10% fetal bovine serum (Qualified FBS, Thermo Fisher Scientific) and 1% penicillin−streptomycin (Thermo Fisher Scientific).

For the quantitative assessment, the cells were seeded (5 × 10^4^ cells/0.5 mL/well) on the untreated and LAB-processed PEEK substrates using a 48-well plate. After culture for 1, 3, and 7 d, cell proliferation and cytotoxicity were evaluated using water-soluble tetrazolium salt (WST)-8 (Cell Counting Kit-8, Dojindo Laboratories, Co., Ltd., Japan) and lactate dehydrogenase (LDH) (Cytotoxicity LDH Assay Kit-WST, Dojindo Laboratories), respectively, according to the manufacturer’s instructions. The optical density (OD) at 450 nm (OD_450_ for WST-8) and 490 nm (OD_490_ for LDH) was measured using a microplate reader (Multiskan FC, Thermo Fisher Scientific).

For the qualitative assessment by microscopic observation, the cells were seeded (5 × 10^4^ cells/1 mL/well) on the untreated and LAB-processed PEEK substrates using a 35-mm dish and cultured for 7 d. The cells were then washed with phosphate buffered saline (PBS), fixed with 3.5% formaldehyde in PBS for 5 min, and permeabilized with 0.5% Triton X-100 for 10 min. After washing with PBS, the cells were treated with 4',6-diamidino-2-phenylindole (DAPI) for nuclear staining at 4°C for a day. The staining solution was prepared with 3 μL of a 1 mg/mL DAPI solution (Dojindo Laboratories) and 500 μL of the 7.5 w/v% albumin Dulbecco's-PBS (-) solution, and shaking the mixture at 37°C for 1 h. The stained cells were observed using a fluorescence microscope (Biorezo BZ-9000, Keyence Corporation, Japan).

### SBF test for preliminary osteoconductivity assessment

The LAB-processed PEEK substrate was immersed in 30 mL of SBF (c-SBF [32]; Na^+^ 142.0, K^+^ 5.0, Mg^2+^ 1.5, Ca^2+^ 2.5, Cl^-^ 147.8, HCO_3_^-^ 4.2, HPO_4_^2-^ 1.0, SO_4_^2-^ 0.5 mM, pH = 7.4 at 36.5°C). After immersion in SBF at 36.5°C for 3 d, the substrate was removed from the fluid and gently rinsed with ultrapure water. Following the SBF test, the apatite-coated substrate was subjected to a tape-detaching test to evaluate the coating adhesion. After drying the substrate, a Scotch mending tape (810-1-12, 3M Company, USA) was attached to a part of the apatite layer formed on the substrate and then detached. The substrate surface before and after the SBF test and the subsequent tape-detaching test were analyzed by SEM, EDX, and thin-film X-ray diffraction (XRD; Ultima IV, Rigaku Co., Japan). The SEM-EDX analysis was performed as described in the section “Physicochemical analysis” using a gold sputter coater (SC-701 Mk Ⅱ, Sanyu Electron, Co., Ltd., Japan) instead of the carbon coater to identify the tape adhesive, in which carbon is the main component, after the tape-detaching test.

### Statistical analysis

The quantitative assessments were carried out using 3 (contact angle measurement) or 4 (solution temperature measurement and cytocompatibility assessment) substrates for each condition. The data are expressed as the average ± (or +) standard deviation. Statistical differences were determined using the Student’s t-test and were considered significant when *p* < 0.05.

## Results and discussion

### Effect of laser fluence

**Fig 2:**
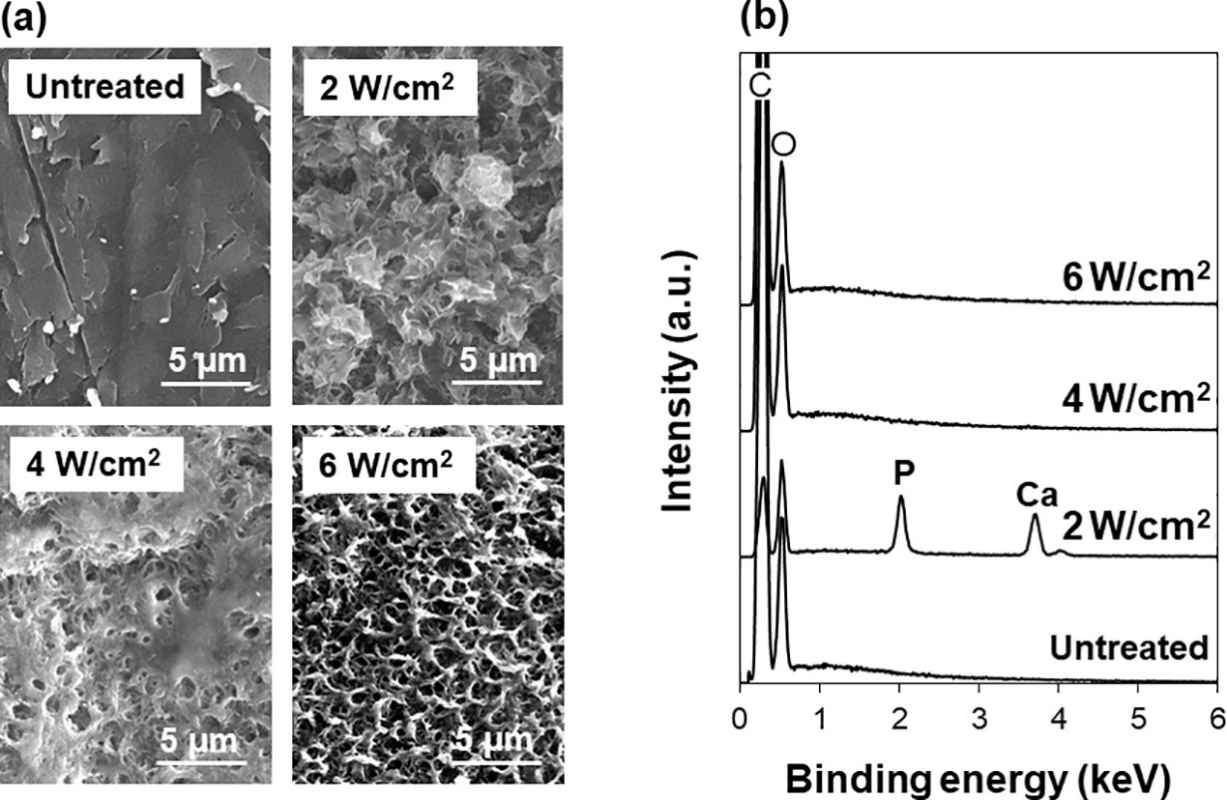
SEM images (a) and SEM-EDX spectra (b) of the surfaces of the untreated PEEK substrate and those after the LAB process at 2, 4, or 6 W/cm^2^ for 30 min. The C peak in (b) is due to the carbon coating prior to the SEM-EDX analysis and the base PEEK substrate.

**Fig 3:**
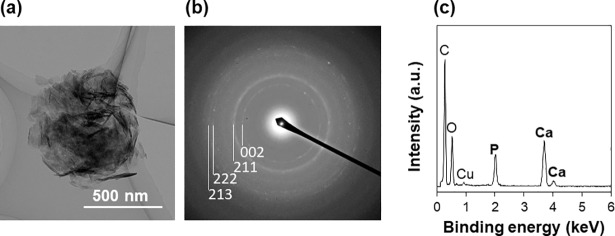
TEM image (a), electron diffraction pattern (b), and TEM-EDX spectrum (c) of the precipitate scraped from the surface of the LAB-processed (2 W/cm^2^, 30 min) PEEK substrate. Rings observed in (b) were indexed to hydroxyapatite (JCPDS No. 09–0432).

The LAB process for CaP coating was applicable to PEEK substrates. CaP formed on the LAB-processed PEEK substrate when the laser fluence was set at 2 W/cm^2^. First, the LAB process was carried out at various fluences from 2 to 6 W/cm^2^ for a fixed irradiation time of 30 min. As shown in the SEM images (Fig 2A), the PEEK substrates present rough surfaces after the LAB process at all the tested fluences. Only on the surface processed at 2 W/cm^2^, Ca and P were clearly detected by SEM-EDX, suggesting the presence of CaP compounds (Fig 2B). We attempted to identify the crystalline phase of the CaP compound by thin-film XRD measurements, but it failed because of the detection limit (see “Before SBF test” in S1 Fig). Thus, we performed TEM analysis for the precipitate scraped from the LAB-processed PEEK substrate. As shown in Fig 3A, the precipitate was an aggregate of nanoparticles. It was reconfirmed by TEM-EDX that the precipitate was composed of Ca and P (Fig 3C). The Ca/P ratio of the precipitate was approximately 1.4. According to its TEM diffraction pattern (Fig 3B), the CaP precipitate contained hydroxyapatite as a crystalline phase. The CaP precipitate did not show a ring (or spot), which is specific to octacalcium phosphate (OCP) (We checked the absence of the strongest diffraction in OCP from (100) planes with interplanar spacing d = 1.878 nm under the analytical condition with a longer camera length.). Similar TEM results were obtained for other precipitates scraped from the same substrate. Judging from the CaP precipitate’s lower Ca/P ratio (~1.4) than stoichiometric hydroxyapatite (1.67), it may contain calcium-deficient hydroxyapatite and/or other CaP phases, such as amorphous CaP and OCP, which are precursors of hydroxyapatite. Their presence, however, was not obvious by the present analytical conditions.

The LAB process at fluences higher than 2 W/cm^2^ was ineffective in CaP coating on PEEK substrates. As revealed by the SEM-EDX spectra (Fig 2B), neither Ca nor P was detected on PEEK surface processed at 4 and 6 W/cm^2^. This result indicates that the surface roughening of these two substrates was not related to CaP formation but instead to laser-induced surface etching. It is reported that there is an appropriate range of fluence for CaP coating in the LAB process for each material. This is because the fluence higher than a certain threshold causes severely destructive surface reactions (melting, ablation, etc.) and depresses CaP formation [33]. It is considered that the fluences of 4 and 6 W/cm^2^ exceeded the threshold for PEEK substrates under the present irradiation conditions. Therefore, the laser fluence was fixed at 2 W/cm^2^ in the following experiments.

### Effect of irradiation time

**Fig 4:**
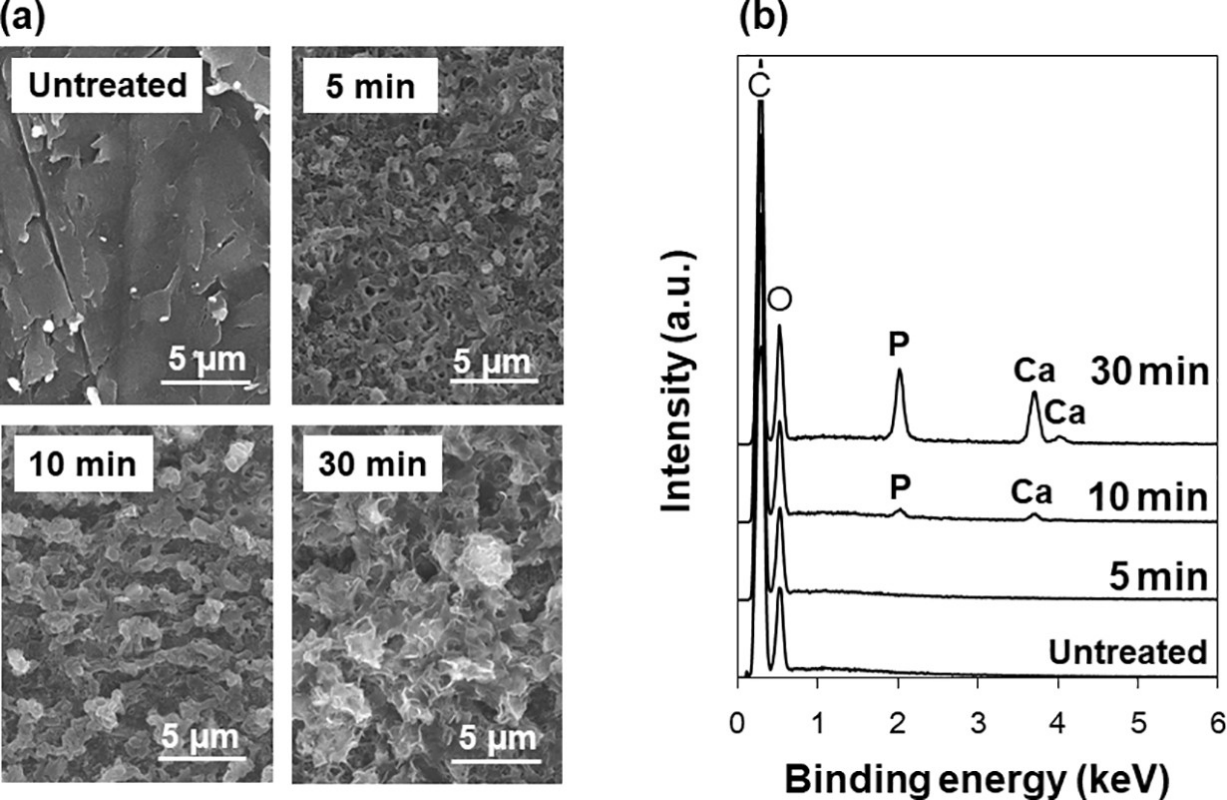
SEM images (a) and SEM-EDX spectra (b) of the surfaces of the untreated PEEK substrate and those after the LAB process at 2 W/cm^2^ for 5, 10, or 30 min. The C peak in (b) is due to the carbon coating prior to the SEM-EDX analysis and the base PEEK substrate.

In the LAB process, CaP started to precipitate on PEEK surface within only 10 min following the surface modification by laser irradiation. As revealed by the time course analysis, the PEEK surface was roughened within 5 min in the LAB process (Fig 4A). At this stage, neither Ca nor P was detected by SEM-EDX anywhere on the surface (Fig 4B). After irradiation for another 5 min (total irradiation time = 10 min), both Ca and P appeared partially on the LAB-processed surface. After irradiation for 30 min in total, both Ca and P were detected on the majority of the LAB-processed surface. Prolonging the irradiation time from 10 to 30 min, the atomic concentrations of Ca and P (averaged from five EDX data at five different positions) on the surface increased by approximately 25 and 22 times, respectively. These results indicate that CaP formed on PEEK surface within 10 min during the LAB process and increased in amount over an irradiation time up to 30 min.

### Area-specific CaP coating

**Fig 5:**
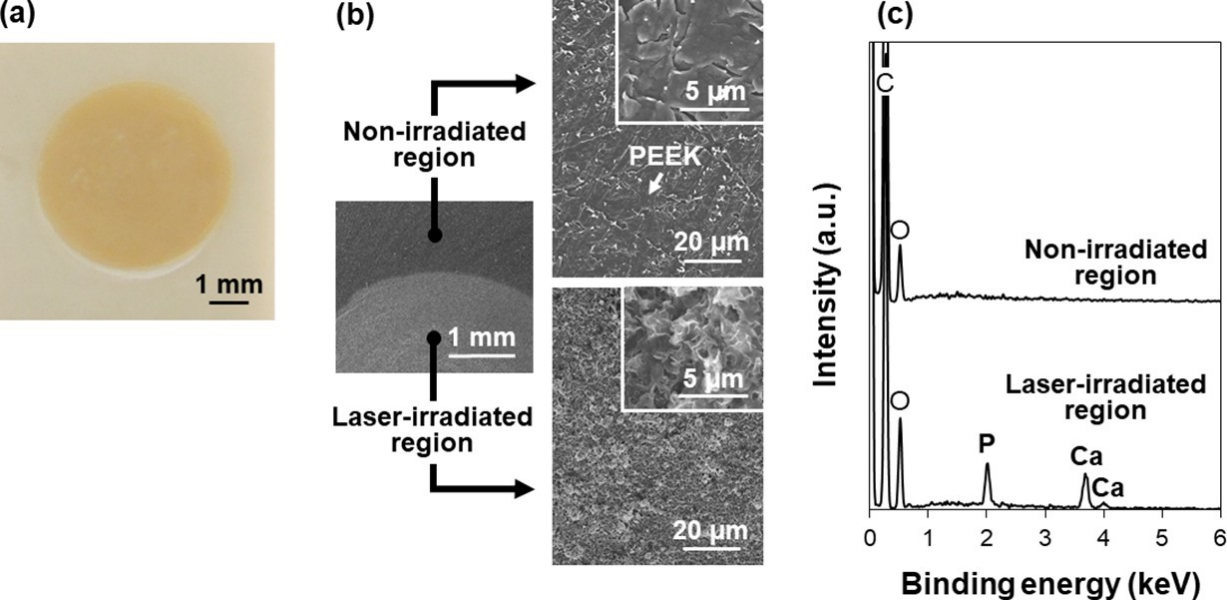
Digital camera image (a), SEM (b) images, and SEM-EDX spectra (c) of the non-irradiated and laser-irradiated regions of the LAB-processed (2 W/cm^2^, 30 min) PEEK substrate surface. The C peak in (b) is due to the carbon coating prior to the SEM-EDX analysis and the base PEEK substrate.

As reported for other materials [25, 27, 33], the LAB process is capable of area-specific CaP coating on PEEK substrates. As depicted in Fig 5, the LAB process induced CaP formation only on the laser-irradiated region (central circular area) without affecting other regions (outside the irradiated area) of the same substrate. No CaP formation on the non-irradiated region was confirmed by the absence of Ca and P peaks in its SEM-EDX spectrum (Fig 5C). These results prove that laser irradiation is essential in CaP formation on PEEK substrates. The role of laser irradiation will be described in the following sections.

### Surface heating by laser irradiation

**Fig 6:**
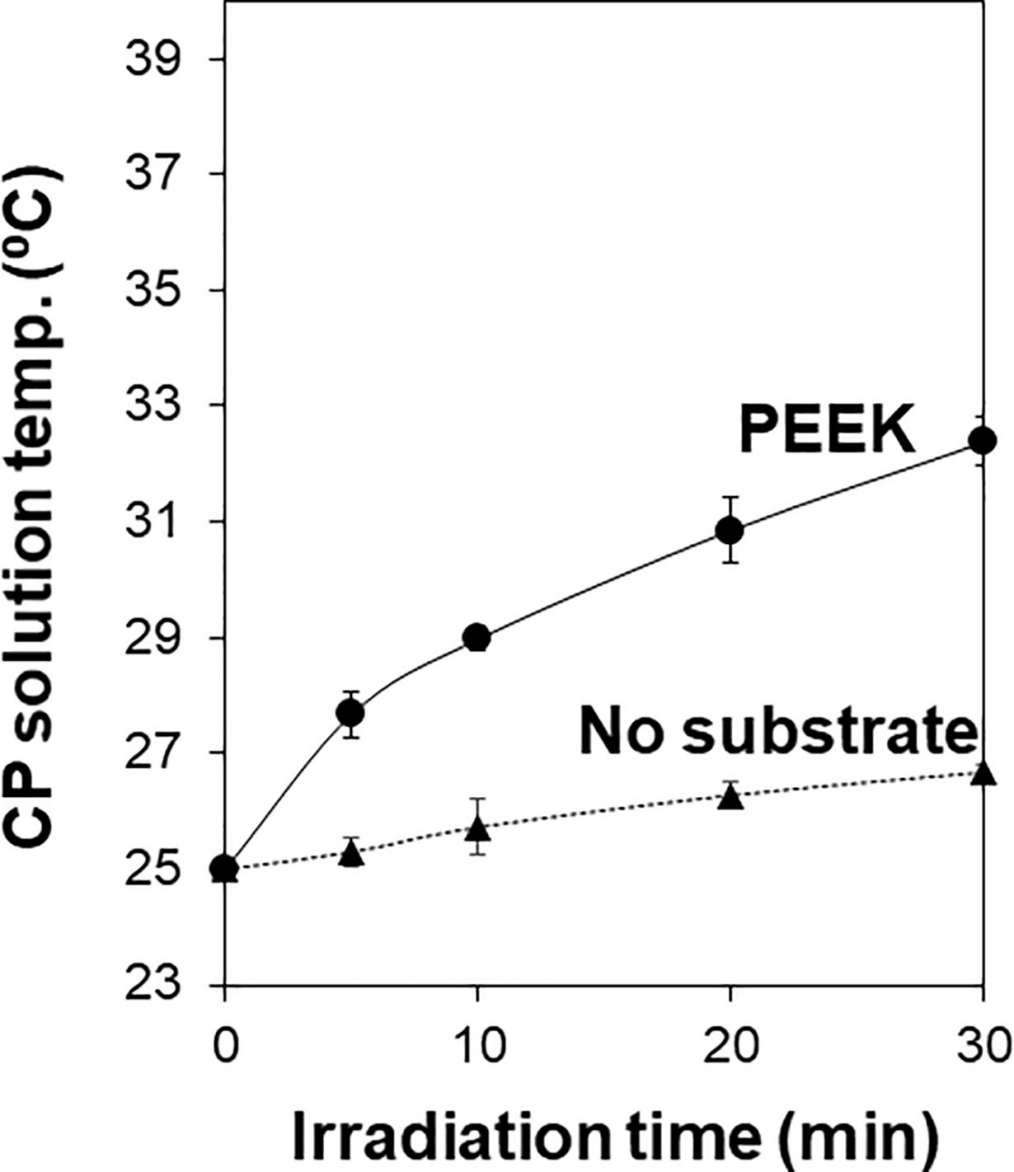
Temperature variations of the CP solution with and without the PEEK substrate during laser irradiation at 2 W/cm^2^ without a temperature-controlled water bath. Data are expressed as the average ± standard deviation (n = 4).

The PEEK substrate absorbed laser light (λ = 355 nm) and was heated on the surface during the LAB process. Light absorption at 355 nm by the PEEK substrate was confirmed by UV-VIS-NIR spectrophotometry (S2 Fig). Surface heating was verified by the first control experiment where the same irradiation process as the LAB process was performed for the PEEK substrate without a water bath. In this experiment, the temperature of the CP solution (25°C at the starting point) elevated with the lapse of irradiation time for the PEEK substrate (Fig 6). The temperature in the CP solution reached approximately 32°C after irradiation for 30 min. Without the substrate, the temperature increase in the CP solution was slight (less than 2°C in 30 min) under the same irradiation condition, since the CP solution scarcely absorbs the light energy at 355 nm [25]. Taken together, the temperature increase in the CP solution with the PEEK substrate should be attributed to laser light absorption by the substrate that leads to the light-to-heat energy conversion at the surface and heat transfer from the surface to the solution.

### Surface modification by laser irradiation

**Fig 7:**
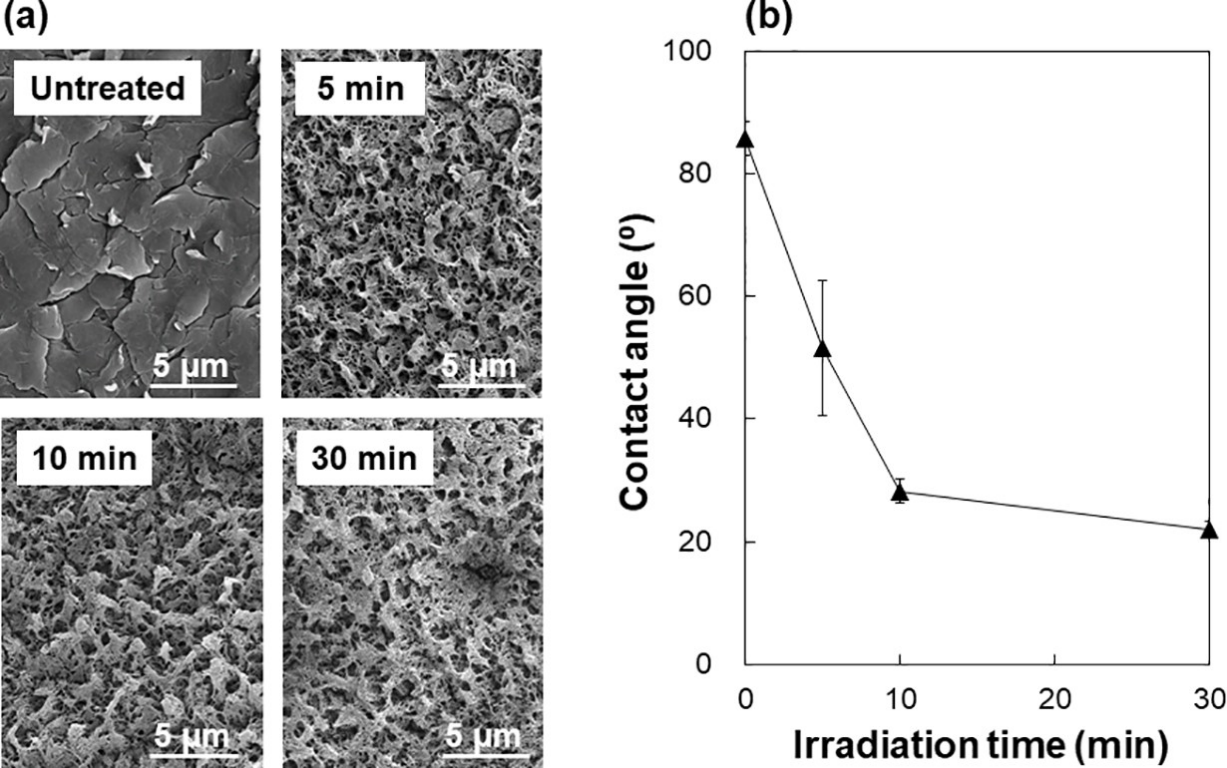
SEM images (a) and contact angles of water droplet (b) of the surfaces of the untreated PEEK substrate and those after laser irradiation in ultrapure water at 2 W/cm^2^ for 5, 10, or 30 min. Data in (b) are expressed as the average ± standard deviation (n = 3).

Laser irradiation caused surface modification of the PEEK substrate, *i*.*e*., surface roughening and wetting, which is basically similar to that observed for other polymers including ethylene-vinyl alcohol copolymer [25] and poly(ethylene terephthalate) [27]. The surface modification was verified by the second control experiment where the same irradiation process as the LAB process was performed in ultrapure water instead of the CP solution. Under irradiation in ultrapure water, the PEEK substrate showed surface roughening (etching) within 5 min (Fig 7A), which is similar to the phenomenon observed in the CP solution (Fig 4A). The contact angle of a water droplet on PEEK surface significantly decreased with increasing irradiation time in ultrapure water (Fig 7B). This result indicates that the water wettability of PEEK surface was increased as a result of laser irradiation. This is most likely due to the laser-induced bond cleavage in PEEK and the formation of polar functional groups, such as hydroxyl, carboxyl, and peroxide, on the surface [34, 35]. These polar functional groups have been reported to function as a CaP nucleating agent in a supersaturated CaP solution [36]. It is natural to assume that even in the CP solution the PEEK substrate should undergo similar surface modification under laser irradiation, triggering heterogeneous CaP precipitation on the laser-irradiated PEEK surface.

### Reaction mechanisms

From the results described above, a putative mechanism of CaP formation in the LAB process can be described based on a non-classical nucleation pathway [37]. The reaction medium (CP solution) in the LAB process was a metastable supersaturated CaP solution that can remain stable for a certain period of time (days to weeks) without inducing homogeneous CaP precipitation. In such supersaturated solutions, prenucleation CaP nanoclusters may exist in a dispersed state without gathering or growing further and behave like solutes [38, 39]. It is known that, in the presence of certain extrinsic surfaces, prenucleation CaP nanoclusters densify heterogeneously at the surfaces to form post-critical amorphous nuclei [40]. According to the results shown in Figs 4 and 5, the LAB process induced surface micro-roughening (etching) within 5 min and CaP nucleation within 10 min specifically at the laser-irradiated PEEK surface. During this time period (within 10 min), water wettability of the laser-irradiated surface was remarkably increased (Fig 7B), which is most likely due to the formation of polar functional groups [34, 35]. It is considered that the laser-irradiated PEEK surface with increased roughness and water wettability had higher affinity with prenucleation CaP nanoclusters than the non-irradiated surface and consequently worked as an active template for CaP nucleation by attracting and densifying these nanoclusters. The thus-formed post-critical nuclei on PEEK surface grew spontaneously with irradiation time (Fig 4) by consuming CaP nanoclusters [38, 39] and ions in the CP solution. Within 30 min, the initially formed CaP in an amorphous phase transformed into thermodynamically more stable hydroxyapatite (Fig 3B) *via* hydrolytic solid˗solid conversion [41]. These sequential reactions (nucleation, phase transformation, growth) were accelerated by laser heating of the PEEK surface and the nearby solution because these CaP compounds are less soluble and transform/grow faster at higher temperatures in neutral solutions [41–44].

### *In vitro* test for cytocompatibility assessment

**Fig 8:**
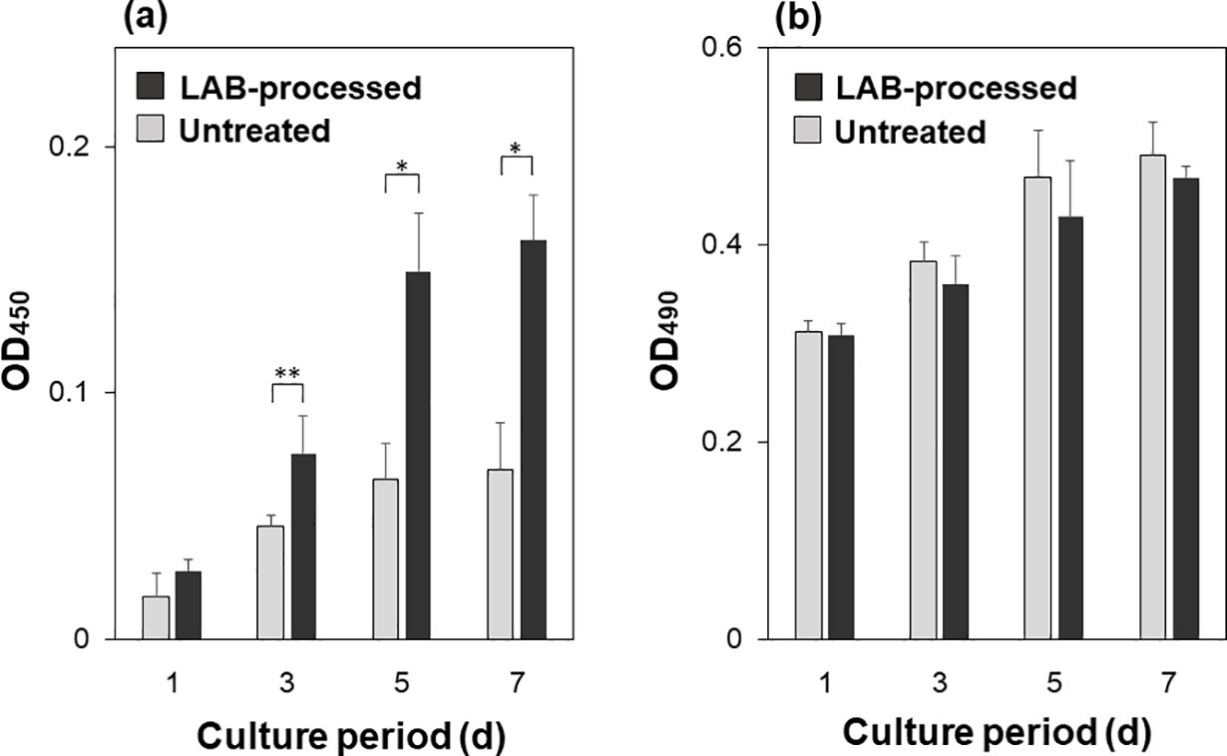
Results of cell proliferation assay (OD_450_: relative to the number of viable cells) (a) and cytotoxicity assay (OD_490_: relative to the concentration of LDH from dead or damaged cells) (b) for the untreated and LAB-processed (2 W/cm^2^, 30 min) PEEK substrates after cell culture for 1, 3, 5, and 7 d. Data are expressed as the average + standard deviation (n = 4, * *p* < 0.05, ** *p* < 0.01).

**Fig 9:**
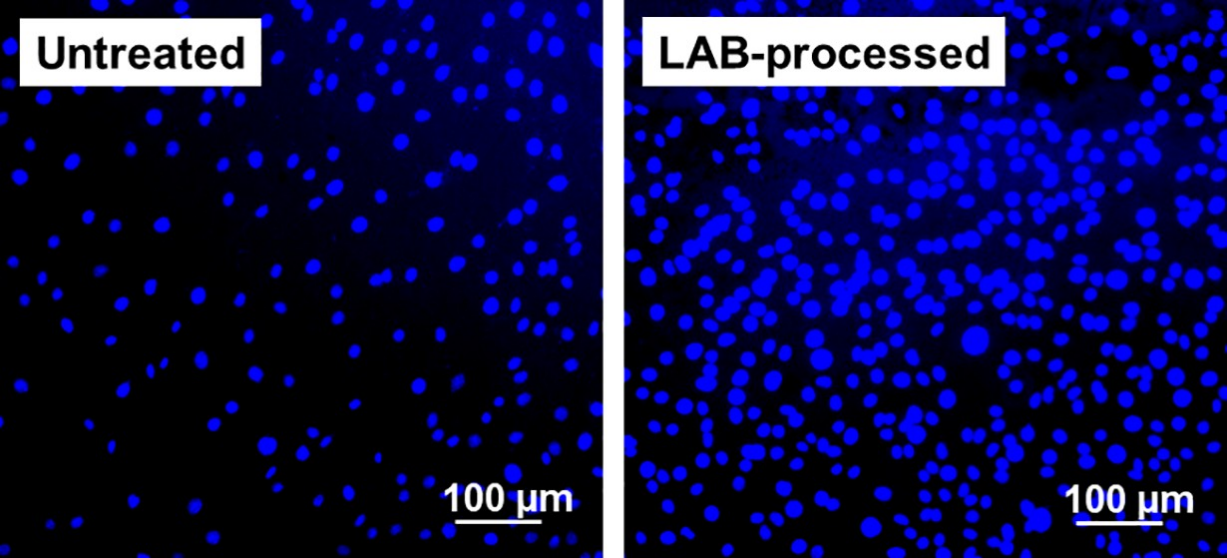
DAPI-stained cells on the untreated and LAB-processed (2 W/cm^2^, 30 min) PEEK substrates after 7 d culture.

Compared with the untreated substrate, the proliferation of osteoblastic MC3T3-E1 cells was enhanced more on the LAB-processed CaP-coated PEEK substrate. As indicated by the WST-8 assay results in Fig 8A, the number of viable cells was comparable (*p* > 0.05) at the initial stage of culture (after 1 d) on the untreated and LAB-processed PEEK substrates. At the later stages (after 3, 5, and 7 d), while cells proliferated on both substrates, the number of viable cells was significantly higher on the LAB-processed PEEK substrate than on the untreated substrate. This difference notably increased after 5 d of culture. To visualize the number density of cells on the substrate after 7 d of culture, they were stained with DAPI (nuclear staining dye) and observed using fluorescence microscopy. As shown in Fig 9, the number density of cells on the LAB-processed PEEK substrate (right) was noticeably higher than that on the untreated substrate (left). According to the LDH assay (Fig 8B), there was no significant difference between the untreated and LAB-processed PEEK substrates with respect to their cytotoxicities. These results suggest that the LAB process improved cytocompatibility of PEEK surface, which agree with our previous results using a different substrate [25].

### SBF test for preliminary osteoconductivity assessment

**Fig 10:**
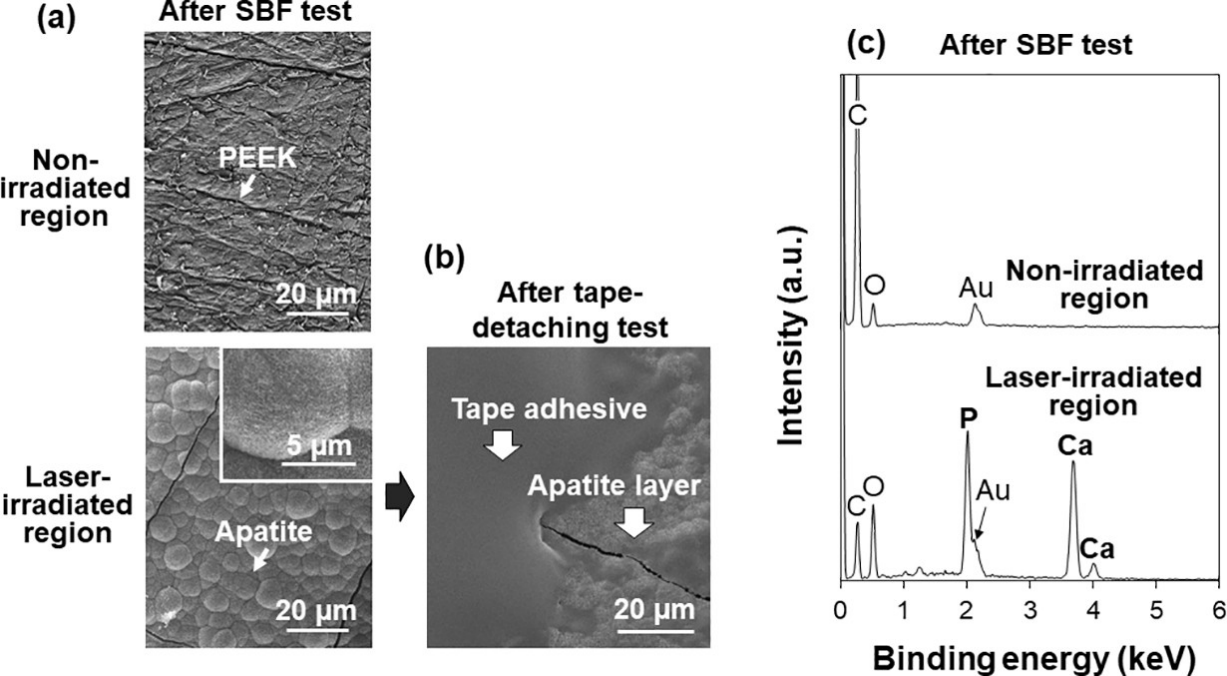
SEM images (a, b) and SEM-EDX spectra (c) of the non-irradiated and laser-irradiated regions of the LAB-processed (2 W/cm^2^, 30 min) PEEK substrate surface after the SBF test (a, c) and after the subsequent tape-detaching test (b). The Au peak in (c) is due to the gold coating prior to the SEM-EDX analysis.

The LAB-processed, CaP-coated PEEK substrate exhibited good apatite-forming ability in SBF, suggesting the osteoconductivity of this material. One concern was the possible risk of CaP dissolution in SBF or in the body because the CaP formed by the wet LAB process was small in amount and should have relatively high solubility compared with sintered hydroxyapatite. This concern for future clinical applications was reduced by the present SBF test, *i*.*e*., the LAB-processed CaP-coated PEEK substrate certainly formed a dense hydroxyapatite layer in SBF within 3 d. As shown in the lower image of Fig 10A, a dense layer was observed on the laser-irradiated region of PEEK surface after undergoing immersion in SBF. This surface layer grown in SBF was composed of CaPs as confirmed by SEM-EDX (lower spectrum in Fig 10C). In the XRD analysis, two diffraction peaks at 26 and 32° ascribed to hydroxyapatite became apparent after the SBF test (S1 Fig). The broadness of these peaks suggests low crystallinity of hydroxyapatite in the layer. Other broad peaks at approximately 23, 29, 33, and 39° occur due to the semicrystalline structure of the PEEK substrate. The apatite formation was observed only in the laser-irradiated region of PEEK surface, while the non-irradiated region of the same substrate was kept intact in SBF (Fig 10A and 10C). This result proves that the untreated PEEK substrate does not possess apatite-forming ability in SBF. This is natural considering the inherent bioinert nature of PEEK [3]. It was found that the initial CaP layer introduced by the LAB process did not dissolve completely in SBF, but instead grew further into a dense and thicker hydroxyapatite layer.

The hydroxyapatite layer grown in SBF adhered to the PEEK substrate so strongly that the layer was not peeled off even after the tape-detaching test. As depicted in Fig 10B, the hydroxyapatite layer withstood the tape-detaching test, and a section of the tape adhesive consisting of C and O (as determined by SEM-EDX analysis) was observed to remain on the layer. Chemical interactions resulting from surface functional groups and the mechanical interlocking effect due to surface roughening might be involved in the strong adhesion between the surface hydroxyapatite layer and the substrate.

Generally, a material having the apatite-forming ability in SBF forms a bone-like hydroxyapatite layer in the body as well, thereby bonding to the surrounding bone tissue through the interfacial hydroxyapatite layer [32]. The LAB-processed PEEK substrate prepared in this study possesses apatite-forming ability in SBF and, therefore, is expected to exhibit osteoconductivity in the body. Further *in vivo* studies are needed to clarify this point.

### Advantages of the LAB process

PEEK is endowed with outstanding chemical durability. Although a number of chemical surface modification techniques have been applied to PEEK to improve its osteoconductivity, the techniques often require a long processing time and/or particular chemicals (toxic in many cases). As demonstrated in this study, the LAB process of only 30 min is effective in CaP coating on PEEK without using any toxic chemicals. In this process, facile one-pot CaP coating is achieved because of the *in situ* laser surface modification effect accompanied by the accelerating effect of laser surface heating on CaP formation. Owing to these effects of laser irradiation, the CaP coating area is confined to the laser-irradiated region of PEEK surface (Fig 5), suggesting that it is possible to functionalize an intended surface region of PEEK by the LAB process while retaining other regions intact.

The LAB process improved the cytocompatibility of PEEK surface with osteoblastic MC3T3-E1 cells; the cell proliferation was promoted on the LAB-processed PEEK substrate compared with the untreated PEEK substrate. Moreover, the SBF test [32] suggested the osteoconductivity of the LAB-processed PEEK substrate, *i*.*e*., the substrate is expected to bond to the living bone tissue through the formation of a hydroxyapatite layer *in vivo*. These are advantages of this material when considering its use in orthopedic and dental implants. In addition, the LAB-processed PEEK substrate may exhibit additional biological functions if certain bioactive substances, such as trace elements and cytokines, are added to the CP solution and are immobilized on the substrate together with CaP. For example, a CaP-coated titanium substrate that was prepared using the CP solution supplemented with fibronectin exhibited enhanced cell adhesion than that prepared using the fibronectin-free CP solution [28]. Another example is a CaP-coated ceramic substrate, which was prepared using the CP solution supplemented with fluoride ions, that exhibited antibacterial activity [45]. In such an approach using the LAB process, PEEK implants with improved and tailored biological functions could be created.

## Conclusions

It was found that the LAB process for CaP coating was applicable to the PEEK substrate. In this process, CaP formed on the PEEK surface after the LAB process of 30 min at an appropriate fluence (2 W/cm^2^). During the LAB process, the laser-irradiated PEEK surface was modified and heated to induce heterogeneous CaP precipitation within 10 min in the CP solution, followed by further CaP growth over the irradiation time (tested up to 30 min). The LAB process improved the cytocompatibility of PEEK surface with osteoblastic MC3T3-E1 cells. Furthermore, the LAB-processed CaP-coated PEEK substrate formed a dense hydroxyapatite layer in SBF, suggesting the osteoconductivity of this material. The present LAB process is capable of rapid and area-specific CaP coating on PEEK, providing a useful new tool to produce osteoconductive PEEK-based implants.

## Supporting information

S1 Fig Thin-film XRD patterns of the surfaces of the LAB-processed (2 W/cm^2^, 30 min) PEEK substrate before and after the SBF test. (TIF)

S2 Fig Absorbance of the untreated PEEK substrate. (TIF)
