# Supplementary figures and images for: Laser-assisted wet coating of calcium phosphate for surface-functionalization of PEEK
Source: PLoS One. 2018 Oct 31;13(10):e0206524. doi: 10.1371/journal.pone.0206524 (PMC6209325; doi:10.1371/journal.pone.0206524)

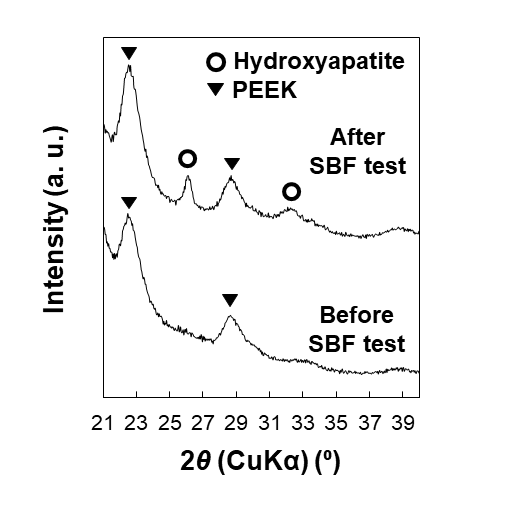

Supplement: S1 Fig — (TIF) [file pone.0206524.s001.tif]

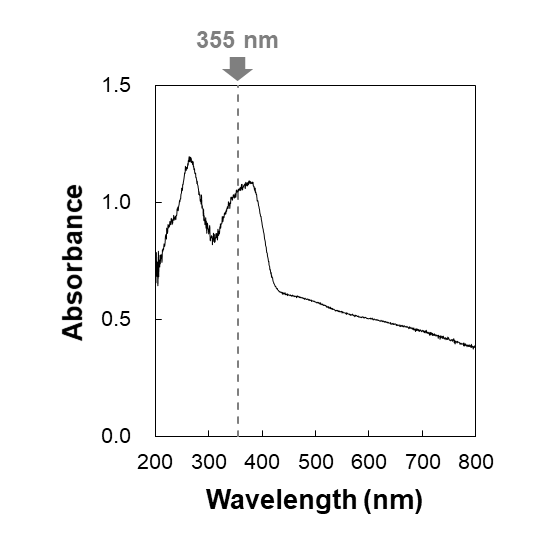

Supplement: S2 Fig — (TIF) [file pone.0206524.s002.tif]
